# Supplementary material for: Conditional chemoconnectomics (cCCTomics) as a strategy for efficient and conditional targeting of chemical transmission
Source: eLife. 2024 Apr 30;12:RP91927. doi: 10.7554/eLife.91927 (PMC11060718; doi:10.7554/eLife.91927)
Supplement: Figure 3—source data 3. [file elife-91927-fig3-data3.zip › Figure 3 source data3 part 2.zip!/Figure 3-Source data 3-part2/1053-1-CRISPResso_Report_mpeqg9da.zip!/CRISPResso_on_1053_3/3b.Insertion_deletion_substitutions_size_hist.pdf]

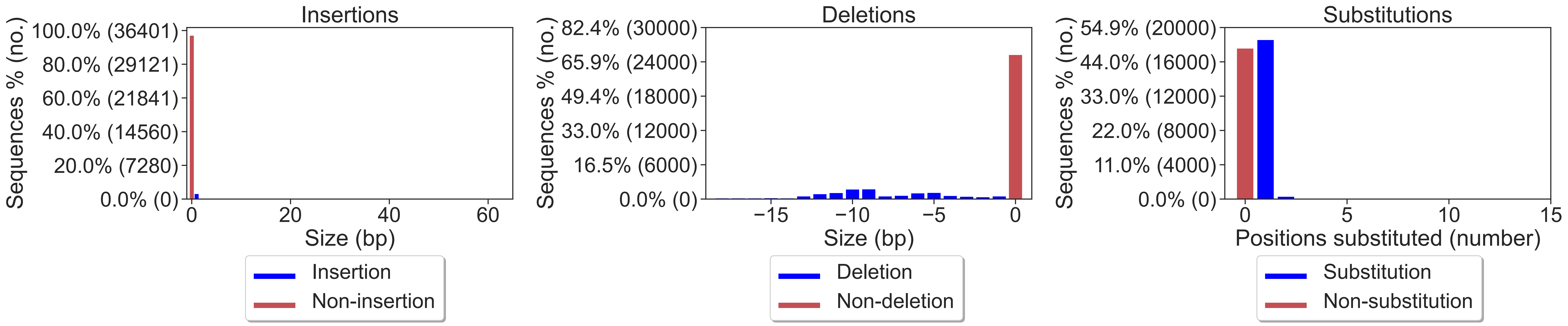

Insertions
Sequences % (no.)
100.0% (36401)
80.0% (29121)
60.0% (21841)
40.0% (14560)
20.0% (7280)
0.0% (0)
0
20
40
60
Size (bp)
Insertion
Non-insertion
Deletions
Sequences % (no.)
82.4% (30000)
65.9% (24000)
49.4% (18000)
33.0% (12000)
16.5% (6000)
0.0% (0)
−15
−10
−5
0
Size (bp)
Deletion
Non-deletion
Substitutions
Sequences % (no.)
54.9% (20000)
44.0% (16000)
33.0% (12000)
22.0% (8000)
11.0% (4000)
0.0% (0)
0
5
10
15
Positions substituted (number)
Substitution
Non-substitution
